# Development and Evaluation of a Quantitative Fluorescent Lateral Flow Immunoassay for Cystatin-C, a Renal Dysfunction Biomarker

**DOI:** 10.3390/s21093178

**Published:** 2021-05-03

**Authors:** Satheesh Natarajan, Maria C. DeRosa, Malay Ilesh Shah, Joseph Jayaraj

**Affiliations:** 1 Healthcare Technology Innovation Centre, Indian Institute of Technology Madras, Chennai 600113, India; malay@htic.iitm.ac.in; 2 Department of Chemistry, Carleton University, Ottawa, ON K1S 5B6, Canada; maria.derosa@carleton.ca; 3 Department of Electrical Engineering, Indian Institute of Technology, Madras, Chennai 600113, India

**Keywords:** quantitative fluorescent immunoassay, non-invasive lateral flow assay, Point-of-Care Cystatin-C test

## Abstract

The diagnosis, prognosis, and control of chronic kidney disease rely on an understanding of the glomerular filtration rate (GFR). The renal clearance of the cystatin-C is closely associated with the GFR. Cystatin-C is a more suitable GFR marker than the commonly used creatinine. General techniques for cystatin-C calculation, such as particle-enhanced turbidimetric and nephelometric assay, are time-consuming and tedious. Here, we propose a rapid, quantitative immunoassay for the detection of cystatin-C. A fluorescence-based lateral-flow kit was developed in a sandwich format by using a monoclonal antibody. A Linear calibration was obtained over the clinical diagnostic range of 0.023–32 µg/mL and the limit of detection (LOD) was 0.023 µg/mL and the limit of quantification (LOQ) was 0.029 µg/mL. Average recoveries from spiked urine samples ranged from 96–100% and the coefficient of variation was less than 4% for both intra and inter-day assays with excellent repeatability. With the comparison with an ELISA kit, the developed kit is highly sensitive, performs well over the detection range, provides repeatable results in a short time, and can easily be used at point-of-care (POC), making it an ideal candidate for rapid testing in early detection, community screening for renal function disorders.

## 1. Introduction

Chronic kidney disease (CKD), ranked 14th among the causes of global death, accounts for 1.2 M deaths every year [1]. In India, the incidence of CKD is increasing rapidly from 26.0 M in 1990 to 115.1 M people in 2017 [1]. The increasing incidence of CKD is primarily associated with lifestyle disorders such as hypertension, cardiovascular disease, hormonal imbalance, and diabetes. CKD is declared as a constant kidney malformation (e.g., GFR < 60 mL/min/1.73 m^2^/urine albumin-to-creatinine ratio (ACR) > 30 mg/g) lasting for more than 3 months [2,3,4]. Staging the clinical diagnosis of CKD, based on GFR, is an important factor for the clinical diagnosis of CKD. Serum creatinine and creatinine clearance rate are the most commonly used parameters for the usual non-invasive determination of GFR. Factors such as muscle mass and protein intake can affect creatinine level, causing unreliable estimation of GFR. Besides, the dynamics of GFR changes tend to lag behind renal activity failure, with a gap named the “creatinine-blind range” that severely limits its use in the early diagnosis and therapy of kidney diseases [5]. Shinde et al. reported that creatinine is an imperfect biomarker for the diagnosis of CKD. Moreover, an increase in the serum creatinine lags (48–72 h) behind the onset of the injury. Besides, serum creatinine is not in a steady-state situation in critically ill patients, leading to the inappropriate estimation of the glomerular filtration [6]. Finally, drugs and many endogenous substances interfere with creatinine quantification [7].

Untreated CKD progress can lead to acute kidney failure (stage V) [8] and mortality. Early detection and treatment are essential as progressive CKD is related to unfortunate clinical outcomes, including end-stage kidney disease (ESKD), cardiovascular disorder, and increased mortality rate. Unfortunately, clinically accepted tests for CKD are cumbersome. They can detect only advanced stages of the disease, i.e., from Stage III to Stage V. Subsequently, there is an urgent need to provide another method for creatinine detection that is clinically more reliable as an early diagnostic marker for CKD patients at stage I and II. [9]. The protein cystatin-C is a 13-kDa cysteine protease inhibitor, freely filtered by the glomeruli with tubular reabsorption and completely removed through the kidneys, which make it a promising endogenous marker candidate to be considered as a filtration marker [10,11]. The concentration of cystatin-C, unlike creatinine, is not influenced by age, gender, protein intake, or muscle mass. Among all the GFR markers, cystatin-C is the only one that can be used for the early detection of tubular cell damage [12]. In patients under 50 years of age, the average level of cystatin-C ranges between 0.52 and 0.92 mg/L and in those above 50 years of age, the normal range is 0.57–1.02 mg/L [13].

Cystatin-C is mostly quantified in urine by using a particle-enhanced turbidimetric immunoassay (PETIA) or nephelometric immunoassay (PENIA) [14,15,16]. Despite high detection sensitivity, automated PETIA and PENIA methods have limited usefulness in daily assessment routines due to their specific instrumentation requirements. ELISA requires costly plate readers and these assays are more error-prone due to their tedious operation, limiting their usage in point of care (POC) diagnostics. Other methods such as photoelectrochemical assays [17], fluorescence [18], near-infrared spectrometry [19], and LC-mass spectrometry-based proteomics [20], microfluidic ELISA [21], electrokinetic microfluidics [22], DNA microarray [23], and microfluidics [24] are alternative methods for the detection of cystatin-C. However, they are too expensive, technically complex, and time-consuming; they require trained personnel and advanced equipment, making them unsuitable for use at the point of need [3,25]. Fluorescence-based lateral flow immunoassay (LFIAs) is widely used for rapid testing at the point of need [26]. Lateral flow immunoassays (LFIA) allow for fast, simple, facile, cost-effective, and on-site diagnosis with good robustness, specificity, sensitivity, and low limits of detection. In this study, we propose a fluorescent-based quantitative lateral flow immunoassay to measure cystatin-C to detect early-stage CKD. This rapid, cost-effective cystatin-C detection offers excellent potential for use in point-of-care and personalized diagnostics, especially in resource-limited settings.

## 2. Materials and Methods

Nitrocellulose membranes (HiFlow135plus) were procured from Merck Millipore (Bedford, MA, USA). Sample and conjugation pad (CF4 and Standard 17) and absorbent pad (CF6) were obtained from Whatman (GE Healthcare, Maidstone, UK). The anti-cystatin-C capturing antibody (Cyst24), the anti-cystatin-C detecting antibody (Cyst28), and recombinant protein cystatin-C (8CY5 Cystatin-C, rec) were obtained from Hytest Ltd., Turku, Finland. Goat anti-mouse IgG H & L, Alexa Fluor™ 647 NHS Ester (Succinimidyl Ester) was obtained from Thermofisher (Shanghai, China). Centrifugal filters were procured from Millipore (Bedford, MA, USA). Sephadex G20 column was obtained from GE Healthcare (Uppsala, Sweden) and PBS, NaOH, Sucrose, BSA, and Tween-20 were procured from Sigma-Aldrich (Munich, Germany).

The key methods used in the development of the cystatin-C LFIA kit are explained in the following sections.

### 2.1. Conjugation of the Alexa fluor 647-mAbs

The monoclonal antibody used for the detection (clone cyst68 for cyst C) was conjugated with a fluorescent organic dye. Alexa Fluor 647 dye (excitation wavelength of AF647 is 633 nm and the emission wavelength is 650 nm) possesses a succinimidyl ester that acts with primary amines on the antibody. Briefly, the detection antibody at 1 mg/mL in 10 mM phosphate solution (pH 7.4) containing 140 mM NaCl (PBS) was reacted with the organic dye (20 molar excess) dissolved in DMSO for 1 h at room temperature. The reaction mixture was separated by Sephadex G20 gel chromatography column by centrifuging at 1300× *g* for 5 min and collecting the labelled antibody in 1× PBS buffer. The synthesized conjugate was mixed with an equal volume of glycerol to the final concentration of 10 µg/mL and stored at −20 °C until use.

### 2.2. Buffer Solutions

The buffer solutions used were as follows: Sample pad buffer (0.1 M PBS, 1% BSA, 0.05% Tween-20, pH 7.4), conjugate pad buffer (0.1 M PBS, 0.05% Tween-20, 20% sucrose and 1% BSA pH 7.4), nitrocellulose membrane blocking buffer (0.1 M PBS, 1% BSA pH 7.4), washing buffer (0.1 M PBS, 0.05% Tween-20 pH 7.4), and assay running buffer (0.1 M PBS, 1% BSA, 0.05% Tween-20, pH 7.4).

### 2.3. Fabrication of Cystatin-C Lateral Flow Immunoassay Membranes

The LFIA membrane strip consisted of four types of pads arranged in a connected manner with limited overlapping. The four membranes were: A sample application pad for the analyte, a conjugate pad made of polyester fiber to hold the conjugated mixture of detection antibody and dye, a nitrocellulose test membrane for the generation of the signals, and finally, the absorbent membrane to promote the capillary flow of the assay. The sample pad was dipped in the sample buffer for 5 min and dried overnight at room temperature. The conjugate pad was immersed in the conjugation buffer and dried overnight; after drying, it was dipped in the conjugation mixture diluted from the original stock (10 µg/mL) to different concentrations (0.5, 1.5, 2 µg/mL) and dried at 37 °C for 1 h. The dye–conjugate solution was diluted in a conjugation buffer. The capture antibody (at 1 mg/mL) and the goat anti-mouse IgG (at 1 mg/mL), both diluted in 1× PBS, were dispensed onto the nitrocellulose membrane using an Easy printer and dried at 37 °C for 1 h. Thereafter, the nitrocellulose membrane was blocked by using 1% BSA with a blocking buffer (50 mL) for 5 min, and washed twice with the washing buffer and dried at 40 °C for 1 h. Finally, the membranes were assembled and cut to a width of 3.2 mm and stored at 4 °C.

### 2.4. Lateral Flow Assay Procedure

In this proposed study, the cystatin-C recombinant protein was spiked into the human urine sample (45 first morning (morning void) urine samples) that had been diluted to the ratio of 1 μL of urine: 74 μL of the buffer. The samples were collected and stored at 4 °C and used for the experiment immediately. The urine samples were collected in sterile containers and used directly (no centrifugation steps). They were prepared in a dilution of cystatin-C of 0, 0.015, 0.03, 0.06, 0.12, 1, 2, 4, 8, 16, and 32 µg/mL in 0.1 M PBS with 1% BSA and 0.1% Tween-20. For the assay development, 75 μL of the spiked sample were dropped in the sample pad packed in the cartridge. The data was acquired from the ImageQuant [27] immunoanalyser (developed by Our Institute, Healthcare Technology Innovation Center, IIT Madras) after 15 min. The signal generated on the nitrocellulose membrane was scanned by the instrument as a two-dimensional pixel map for quantifying the biomarker. The fluorescence signals at the test line and control line were used to calculate the volume ratio (T/C volume ratio). The entire assay was done in triplicate, and the corresponding mean volume ratio (V_R_) value was plotted vs. the cystatin-C concentration to get the calibration curve.

The fluorescent pixel map was processed using the NI LabView software mentioned in our previous work by Malay et al. [27,28]. The calibration equation for the Volume Ratio (VR) calculation was performed as mentioned by image-analytics techniques, to identify the reaction kinematics from a sequence of images. It is also used to identify the development of the test and control lines, track the reaction progress, identify the stabilization of the reaction, and calculate the test and control line areas and area ratios at a stable time to provide repeatable results [29]. To calculate the Ab concentration (mg/mL), we used the following: (A_280_ − (A_650_ × 0.03) × dilution factor/11,000, where 11,000 is the molar extinction coefficient of cystatin-C and 0.03 is a correction factor to account for the absorption of the dye at 280 nm.

### 2.5. Determining the Limit of Blank (LoB) and Limit of Detection (LoD)

The LoB and LoD were estimated according to the CLSI EP-17A2 guidelines [30]. For LoB, the 95th percentile of 50 samples with no cystatin-C was determined. Six samples with the lowest cystatin-C concentration were measured in triplicate for the consecutive five days to calculate the LoD. LoD was calculated as LoD = LoB +1.65 SD samples, where [31]

(1) $$SD\;samples=\frac{\sqrt{{\left(SD1\right)}^{2}+{\left(SD2\right)}^{2}+{\left(SD3\right)}^{2}+{\left(SD4\right)}^{2}+{\left(SD5\right)}^{2}+{\left(SD6\right)}^{2}}}{6}$$

The glomerular filtration rate (GFR) was calculated by using the cystatin-C formula given by Stevens et al. [32]:

GFR = (78/cystatin-C) + 4(2)

### 2.6. Recovery

The recovery was assessed by adding different concentrations of cystatin-C standards to the urine samples. Recovery was expressed as the percentage measurement of the amount added, as calculated from Equation (3)

(3) Recovery(%)=(measure concentration/spiked concentration) × 100

### 2.7. Stability Test

In ten aluminium foil pouches, three strips of each concentration of the cystatin-C were prepared. Five pouches were kept at 4 °C for 0 and 90 days and the remaining were stored at 37 °C for 0, 2, 6, and 8 days. All the pouches were taken from the oven and the fridge on days 0 and 90; the strips were tested for determining the cystatin-C concentration.

### 2.8. ELISA

Human Cystatin-C ELISA Kit (ab119589) KIT was purchased from Abcam, UK. The procedure was followed according to the manufacturer’s protocol.

### 2.9. Statistical Analysis

All quantitative data of the assay were calculated by Graph Pad Prism 6.0 (Graph Pad Software, La Jolla, CA, USA) for determining its sensitivity (Se) and specificity (Sp). Each parameter was calculated using a 95% confidence interval (CI) and the (CoV) of COI values was also analyzed for the assay data. The values for the (LOB) and (LOD) were obtained by using the 1.645 × SD of the negative samples and LOD = LOB + 1.645 (SD lower level of the sample), respectively [33]. A urine sample that was tested negative for cystatin-C in ELISA was used for the experiment.

## 3. Results and Discussion

### 3.1. Principle of the Quantitative Fluorescent Lateral Flow Immunoassay

**Figure 1:**
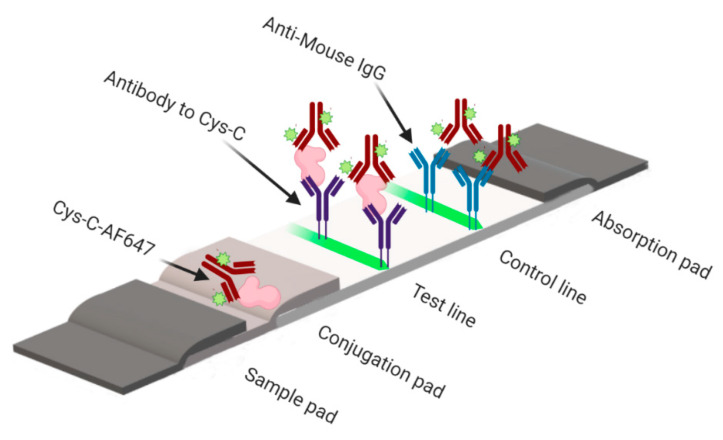
Schematic presentation of fluorimetric lateral flow immunoassay (LFIA) for the determination of cystatin-C. In the presence of cystatin-C, a fluorescence signal from the AF647-labelled cystatin-C antibody can be detected at the test line. See Supplemental Figure S1 for sample images.

The principle of a sandwich fluorescent LFIA is illustrated in Figure 1. The cystatin-control is diluted at different concentrations from 0.015 to 32 µg/mL in 0.1 M PBS, 0.1% Tween and spiked with the urine sample. If there is no cystatin-C in the diluted control, the AF647–cystatin-C mAb will be captured by the capture antibody in the test line and the goat anti-mouse IgG in the control line. If cystatin-C is present in the assay, this target will react with the AF647-cystatin-C conjugates in the conjugation pad and move further to react with the test line capture antibody to form a sandwich of AF647–cystatin-C–analyte–capture antibody while unreacted conjugates will move further. There will be a positive correlation between the presence of the fluorescence intensity in the test line and the analyte content in the sample, which can be used to quantify the analytes present in the sample.

### 3.2. Optimization of the Parameters for the Lateral Flow Immunoassay

The fluorescent lateral flow immunoassay was developed and optimized to estimate the cystatin-C value to calculate the glomerular filtration rate. Table 1 shows the parameters that were optimized in the LFIA. The parameters that most influenced the LFIA performance were the concentration of the capture antibody in the nitrocellulose membrane, the amount of conjugate–antibody mixture in the conjugation pad, pre-treatment of sample pad, and conjugation pad blocking of the nitrocellulose membrane.

**Figure 2:**
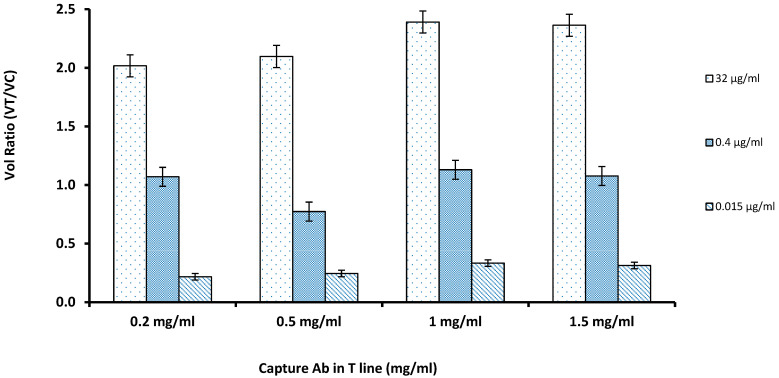
The optimal concentration of capture antibody on the test line was evaluated by comparing Volume Ratio with varying amounts of the capture antibody coated on the Test Line against the three concentrations of the cystatin-C (32, 0.4, and 0.015 µg/mL); 1 mg/mL was chosen to maximize the volume ratio while minimizing antibody use (*n* = 3).

For coating the capture antibody at the nitrocellulose membrane, the capture antibody solution was diluted into concentrations of 0.2, 0.5, 1, 1.5 µg/mL. The amount of capture antibody on the test line has a large influence on the sensitivity of the test strip assay. If the concentration of the antibody is too high, it could lead to nonspecific adsorption. The results showed that inadequate amounts of antibodies in the nitrocellulose membrane led to the low volume ratio and that increasing the antibody concentration improved the V_R_ of the LFIA strip. According to the results shown in Figure 2, it was concluded that 1 mg/mL of the capture antibody in the nitrocellulose membrane was found to be the optimal concentration, thus the remaining experiments followed the same level. As the concentrations were increased from 0.5 to 1.5 mg/mL, the increase in the VR was not significant at 1.5 mg/mL (*p* > 0.05); hence, 1 mg/mL was finalized.

**Figure 3:**
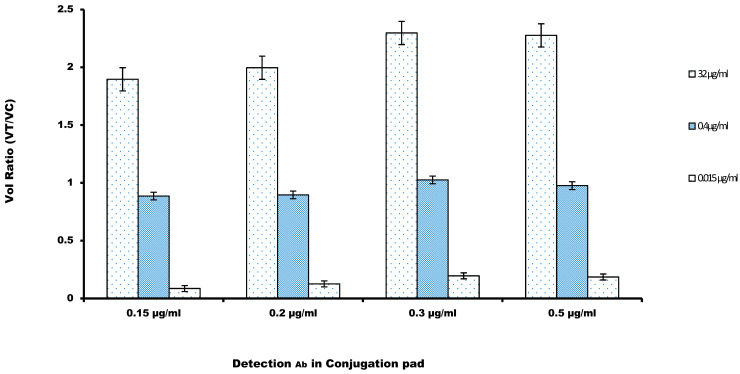
Volume Ratio was calculated against the amount of the detection antibody in the conjugation pad. Three concentrations of the cystatin-C (32, 0.4, and 0.015 µg/mL) were considered for the determination of the optimal antibody concentration (*n* = 3). A value of 0.3 µg/mL was chosen to maximize the volume ratio.

Glynou et al. [34] recorded that the appropriate amount of the detection antibody, labelled with the fluorochrome dye, can increase the sensitivity of a lateral flow immunoassay. The amount of anti-cystatin-C antibody coupled with fluorochrome AF647 was screened, mainly by diluting the conjugation mixture to 0.15, 0.2, 0.3, and 0.5 μg/mL. Then, the prepared AF647-mAb was run on the LFIA membranes. The volume ratio (V_R_) recorded in Figure 3 showed that there was a rise in the volume ratio (V_R_) with the increase in the amount of Alexa fluor labeled mAb and a peak was recorded at approximately 0.3 μg/mL, and then, it decreased gradually. Based on this observation, the amount of labelled antibody of 0.3 μg/mL was chosen as the optimum amount for the strip.

Lateral flow immunoassay performance was evaluated in the sense of pre-treatment given to the sample pad. Many reports showed that when the sample pad was pre-treated with a dilution buffer with surfactants, such as Tween 20 or PEG, it increased the sample flow, eliminated the background, and increased the sensitivity and stability of the LFIA [35]. In our study, we evaluated the sample running buffer by testing the buffer with the different concentrations of Tween-20 ranging from 0.01 to 0.15% and finally, a pre-treatment solution consisting of 0.1% Tween-20 was selected (Supplementary Figure S2).

### 3.3. Effect of Nonspecific Interaction on Assay Quality

**Figure 4:**
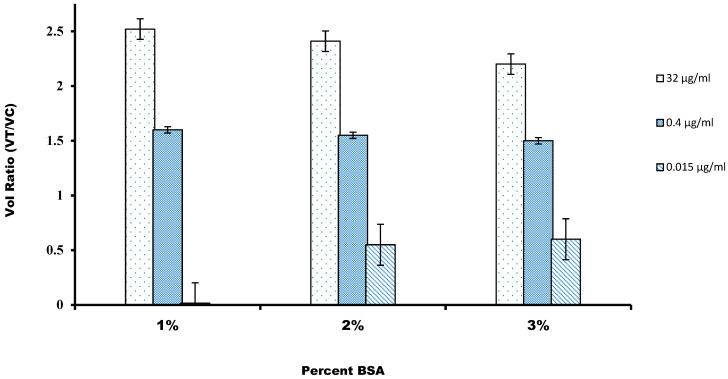
The volume ratio was calculated against the percentage of BSA used for the blocking of the nitrocellulose membrane. A value of 1% was chosen to maximize the volume ratio.

For the nonspecific binding, mainly to avoid false-positive results, we characterized the different blocking solutions for the nitrocellulose membrane. For this, we used five different blocking buffer compositions, containing BSA, a commonly used blocking agent, and the surfactant Tween-20 was used to reduce the hydrophobic effect and surface tension of the sample buffer, and the reaction was carried out at pH 7.4. Figure 4 shows that the highest intensity of volume ratio was recorded on the test when the nitrocellulose membrane was blocked with a blocking buffer. The reason might be that higher BSA concentration might saturate the high surface area of the nitrocellulose fiber network, which blocks nonspecific binding of components of the immunoassay on the membrane surface. The very basic mechanisms by which molecules interact with the nitrocellulose membrane’s surface are primarily dependent on the surface’s hydrophilic or hydrophobic nature [36]. Nonspecific binding of analytes on a nitrocellulose surface includes analytes that might have bound outside the detection sites or other substances bound to the detection site. Along with that, the conjugated mixture might interfere with the cellulose fiber in the conjugation pad or might be trapped physically inside the fiber structure of the membrane. The adsorption behavior of BSA on the hydrophilic and hydrophobic surfaces has been examined and it has been reported that BSA leads to the complete coverage on a hydrophilic surface in a PBS buffer of pH 7.4. It was confirmed by the reaction of the phosphate–surface–BSA complexes [36]. It has also been suggested that the interaction might be between positively charged BSA with the negatively charged nitrocellulose membrane.

**Figure 5:**
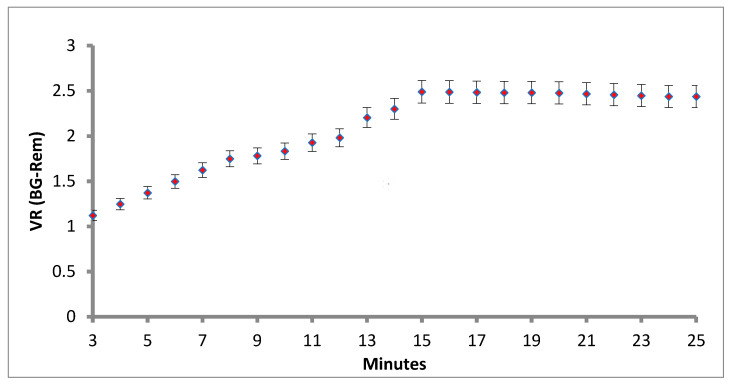
Standardization of the lateral flow assay reaction time.

Another critical parameter is the assay running time for the LFIA, because the immunoreaction time is a key element for developing the fluorescence intensity in the strip test. In this work, the volume ratio (V_R_) of T line to C line is illustrated in Figure 5. Standardization of the lateral flow assay reaction time of the LFIA increased with the assay time. We used 20 µg/mL concentrations of cystatin-C standard and each concentration was done in triplicate. The results are shown in Figure 5. At the concentrations of 20 µg/mL, the V_R_ ratio increased rapidly in the first 15 min and then changed only slowly after 15 min. Finally, we selected 15 min as the most suitable incubation time for further experiments (Figure 5).

### 3.4. Analytical Validation and Functional Detection Limit

**Figure 6:**
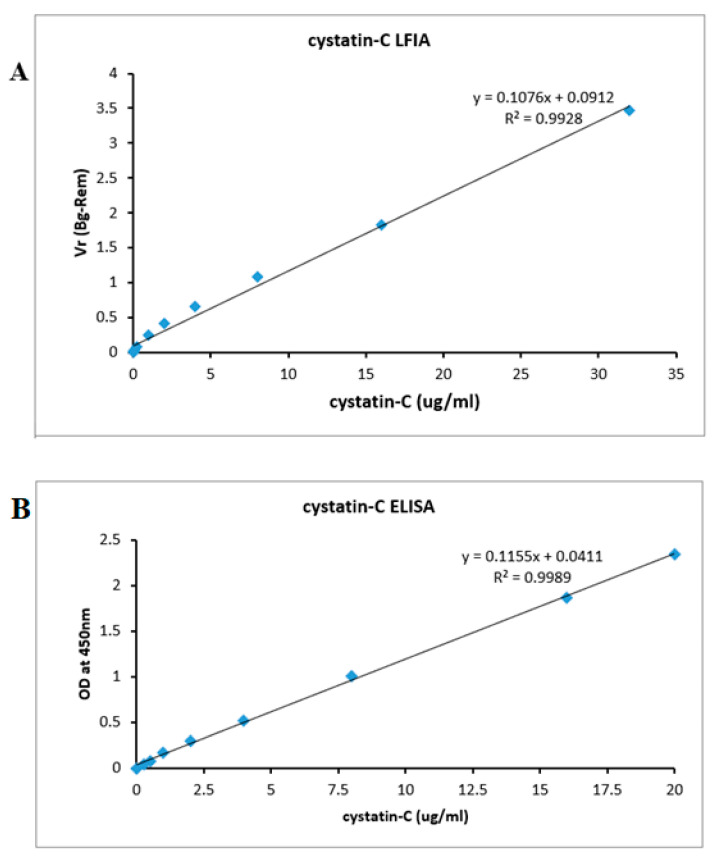
(**A**) The calibration plots of the LFA for cystatin-C was constructed by plotting the V_R_ (BG-Rem) ratio × various concentrations of the cystatin-C standard solutions. Data represent the average of three measurements. Note that Figure S4 shows the four-parameter logistic fit for these data. (**B**) The calibration plots of the ELISA for cystatin-C was constructed by plotting optical density at 450 nm × various concentrations of the cystatin-C standard solutions. Symbols are representative of the different trials.

Analytical validation was studied by spiking the cystatin-C recombinant protein samples into urine samples, whose initial cystatin-C concentrations were 0.015, 0.03, 0.06, 0.12, 0.21, 1, 2, 4, 8, 16, and 32 μg/mL. The calibration curve was assessed by plotting the observed cystatin-C concentration against the V_R_ ratio (Figure 6). The assay response was linear (r = 0.9928) throughout the measured cystatin-C concentration range (Y = 0.1076x + 0.0912, *n* = 10). The calculated limit of quantification (LOQ) was 0.023 µg/mL and the limit of detection (LOD) was 0.029 µg/mL. Table 2 shows some of the commonly used detection techniques for the LOD of cystatin-C detection. The present method compared favorably with the other methods and with the ELISA run with identical sample preparation. Furthermore, the proposed LFIA also achieved excellent analysis performance, meeting the LOD requirements of cystatin-C point-of-care test detection.

### 3.5. Precision

To determine the within run and between run variances, we analyzed five replicates from the concentration between 0.015 and 32 µg/mL on each of the six days. The precision was calculated using the one-way analysis (ANOVA). Data from the replicates for the consecutive six days are compiled in Table 3, with the raw data and error per cent found in Table S2 and Figure S2, respectively. The within run variance averaged 0.6% and between runs, the variance does not exceed 3.5% [39]. The intra-assay was performed within 1 day with ten replicates at each spiked concentration, and the inter-assay was performed every 3 days for the continuous 10 days, with three replicates at each spiked concentration.

### 3.6. Recovery Study

The assay recovery was determined by the measured concentration of cystatin-C divided by the theoretical concentration. Control urine samples were spiked with three various concentrations of cystatin-C standard samples with the low, medium, and high concentration, respectively (0.03, 1, 16, 32 µg/mL), for analysis, and the recovery rates of the four selected samples were 92, 117, 102, and 105%, respectively (Table 4).

### 3.7. Stability

The accelerated ageing test was done to evaluate the stability of the strips by storing the strips at 37 °C for 7 days. For the determination of the stability, control strips were stored at 4 °C until the end of the experiment. On the other hand, experimental strips were kept at 37 °C until the completion of the experiment and then transferred to a 4 °C fridge. As shown in Supplementary Table S3, it was observed that after 7 days, the CV of the strips (32, 0.4, 0.015 µg/mL) were 1.7%, 3.50%, and 3.50%, respectively, and it showed no significant changes. According to the Arrhenius equation [40], the stability (longevity of the test strips from their date of manufacture to the date of use) of the LFIA system is valid for 12 months of storage at 4 °C. These strips can theoretically be stored at 4 °C for a minimum of one year without much change in the volume ratio.

### 3.8. ELISA

To investigate the potential clinical application of our Quantitative-lateral flow immunoassay, we compared its analytical performance with that of a commercially available ELISA assay kit (Abcam, UK). Eleven different concentrations (0, 0.3, 0.5, 1, 2, 4, 8, 16, and 20 μg/mL) of cystatin-C were prepared and tested using both methods. As shown in Figure 6, the minimum detectable concentration of cystatin-C using the commercial assay kit is the limit of detection (LOD) which was 0.0191 µg/mL and the limit of quantification (LOQ) which was 0.013 µg/mL (Figure 6B); this is more or less equal to the minimum detection measured by our Quantitative-based lateral flow assay (0.023 ng/mL). The results suggest that our quantitative lateral flow immunoassay is suitable for the diagnosis of renal dysfunction infections.

## 4. Conclusions

A cystatin-C antibody-based lateral flow immunoassay was successfully developed, optimized, and verified. The calculated limit of quantification (LOQ) was 0.029 µg/mL and the limit of detection (LOD) for cystatin-C standard solutions spiked in urine samples was 0.023 µg/mL. The precision of the assay determined by the CV from the intra-assay and inter-assay was less than 10%, which comes under the acceptance level for the LFIA. The cystatin-C LFIA strips showed stability for one year at 4 °C. The stability test showed that, after a week at 37 °C, the volume ratio of the strips had not significantly changed, which means that the strips are stable at 4 °C for one year. The recovery level of the LFIA for the cystatin-C in the spiked urine samples was almost 100% for 10 µg/mL of cystatin-C. This new LFIA for cystatin-C shows increased sensitivity. The comparative study was done with the ELISA KIT to identify the significance of the fluorescent LFIA and demonstrate that the agreement with our kit cystatin-C rapid test card meets the requirements of clinical applications [39].

## Figures and Tables

**Table 1:** Optimization of the experimental conditions for the lateral flow detection assay.

Experimental Condition	Value Tested	Optimal Value
Capture antibody on T line	0.2, 0.5, 1, 1.5 mg/ml	1 mg/mL
Detection antibody on conjugation pad	0.15, 0.2, 0.3, 0.5 µg/mL	0.3 µg/mL
BSA percentage in the running buffer	3%, 2%, 1%	1%

**Table 2:** A comparative study was carried out to identify the significance of the fluorescent LFIA with the reported works of literature and the current work.

S.No	Detection Methods	Limit of Detection (µg/mL)	Detection Time (Min)	References
1	PENIA (Siemens)	0.53	6	[37]
2	PETIA (COBAS)	0.47	10	[37]
3	PETIA (Genzyme)	0.61	10	[37]
4	SPRI sensor	0.09	10	[38]
5	SPMWE sensor	0.006	10	[3]
6	Fluorescent LFIA	0.023	15	Present study

**Table 3:** The precision of the LFIA for detecting cystatin-C co-spiked samples (*n* = 4).

Sample	Mean	Inter Assay (%CV)	Intra Assay (%CV)	Total	CV%
1	0.15	0.01	0.152	0.162	2.13
2	0.03	0.245	0.325	0.57	2.25
3	0.06	0.125	0.452	0.577	2.35
4	0.12	0.325	0.524	0.849	3.12
5	1	0.345	0.527	0.872	3.21
6	2	0.345	0.538	0.883	3.41
7	4	0.521	0.652	1.173	3.5
8	8	0.521	0.650	1.171	3.45
9	16	0.510	0.649	1.159	3.46
10	32	0.521	0.642	1.163	3.47

**Table 4:** Recovery study of the Fluorescent LFA for detecting cystatin-C (*n* = 4).

Theoretical Concentration (µg/mL)	Measured Concentration (µg/mL)	Recovery (%)
0.03	0.026	92
1	1.37	117
16	16.5	102
32	32.6	105

## Data Availability

Not applicable.

## Supplementary Materials

The following are available online at https://www.mdpi.com/article/10.3390/s21093178/s1: Figure S1. Sample images of the fluorimetric LFIA strips. Figure S2.The graph depicts the error percentage for the cystatin C plotted against the value cystatin C. Figure S3. Volume ratio was calculated against the Tween concentration used in the running buffer. Figure S4. The calibration plots of the LFA for cystatin-C fitted with a 4 parameter logistic model for comparison. Table S1. Raw data for the coefficient of variation for the inter-assay. Table S2. Raw data for the coefficient of variation for the intra-assay. Table S3. Stability test for the cystatin C lateral flow strips.
