# Supplementary material for: Development and Evaluation of a Quantitative Fluorescent Lateral Flow Immunoassay for Cystatin-C, a Renal Dysfunction Biomarker
Source: Sensors (Basel). 2021 May 3;21(9):3178. doi: 10.3390/s21093178 (PMC8125764; doi:10.3390/s21093178)
Supplement: Supplementary file 1 [file sensors-21-03178-s001.zip › sensors-1166556-supplementary.pdf]

Electronic Supplementary Material

Development and evaluation of a quantitative fluorescent lateral flow immunoassay for cystatin C, a renal dysfunction biomarker.

Satheesh Natarajan [1*], Maria DeRosa [2], Malay Ilesh Shah[1], and Joseph Jayaraj [3*]

[1] Healthcare Technology Innovation Centre, Indian Institute of Technology Madras, Chennai 600113, India; malay@htic.iitm.ac.in

[2] Department of Chemistry, Carleton University, Ottawa, ON K1S 5B6, Canada; maria.derosa@carleton.ca

[3] Department of Electrical Engineering, Indian Institute of Technology, Madras, Chennai 600113, India

***** Correspondence: satheesh@htic.iitm.ac.in (S.N.); jayaraj@htic.iitm.ac.in (J.J.)

Contents:

Figure.S1

Sample images of the fluorimetric LFIA strips. 3

Figure S2

The graph depicts the error percentage for the cystatin C plotted against the value of cystatin C 3

Figure. S3
Volume ratio was calculated against the Tween concentration used in the running buffer 4

Figure. S4
The calibration plots of the LFA for cystatin-C fitted with a 4 parameter logistic model for comparison 4

Table S1 Raw data for the co-efficient of variation for the inter-assay 5

Table S2 Raw data for the co-efficient of variation for the intra-assay 5

Table S3 Stability test for the cystatin C lateral flow strips 5

A)

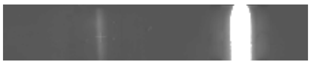

B)

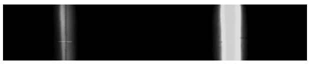

C)

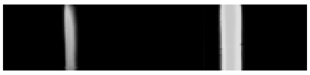

D)

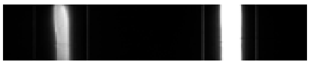

Figure.S1: Sample images of the fluorimetric LFIA strips. Test line is on the left and the control line is on the right. a) 0.013 µg/ml b) 2 µg/ml c) 16 µg/ml, and d) 32 µg/ml.

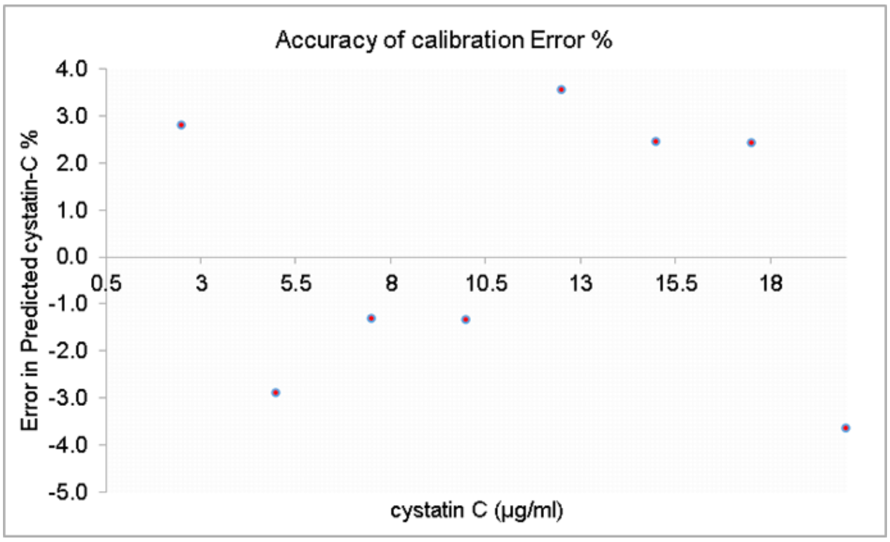

Figure. S2 The graph depicts the Error percentage for the cystatin C plotted against the value cystatin C. The error percentage of the assay. Both the blank solutions and spiked sample containing the different concentration of the analyte were prepared for the by lateral flow immunoassay. The acceptable level of error is 4% for cystatin C.

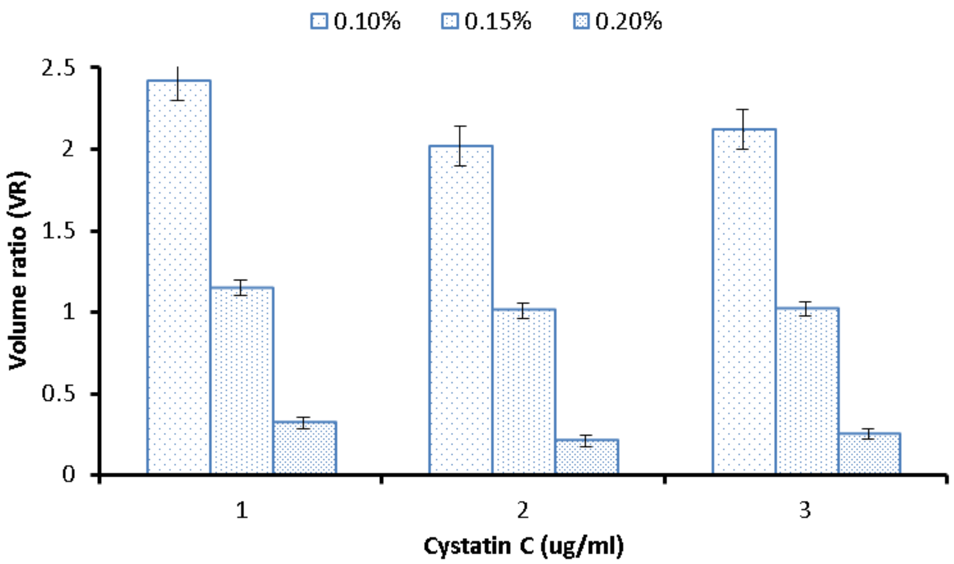

Figure. S3: The graph depicts the Volume Ratio was calculated against the amount of the Tween in the assay buffer, three concentration of the cystatin C (20, 10, and 2.5 µg/ml) were taken into consideration for the determination of the antibody concentration and was finalized based on the data. (n = 3)

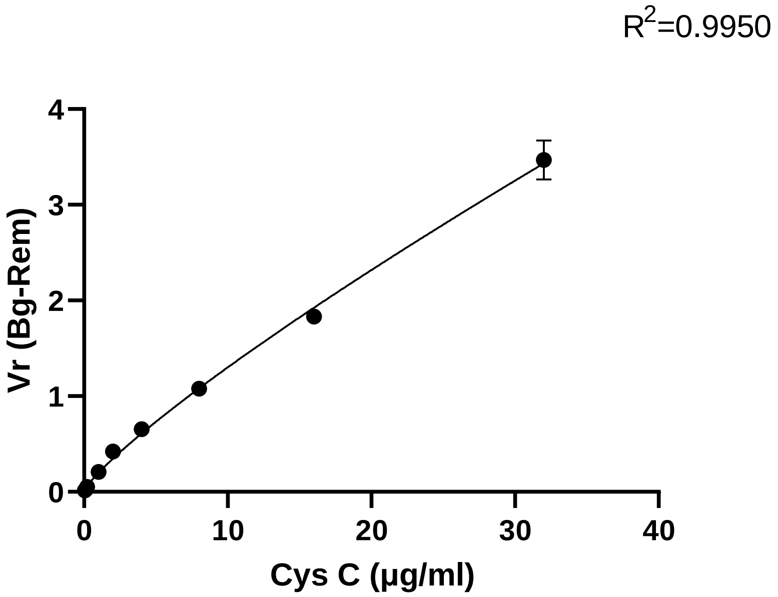

Figure. S4: The calibration plots of the LFA for cystatin-C fitted with a 4 parameter logistic model for comparison

Table S1 Raw data for the co-efficient of variation for the inter-assay

I	2	3	4	5	6	7	8	9	10	Total
0.0006	0.0156	0.0112	0.0186	0.0146	0.0195	0.0146	0.0186	0.0186	0.0145	0.0142
0.2492	0.2486	0.2895	0.1246	0.2957	0.2985	0.2100	0.2154	0.2215	0.2130	0.2414
0.1242	0.1465	0.1012	0.1986	0.1200	0.1651	0.1012	0.1978	0.1210	0.1986	0.1474
0.3885	0.3214	0.3120	0.2987	0.3125	0.2978	0.3214	0.2979	0.3125	0.2945	0.3157
0.3885	0.3214	0.3220	0.3487	0.3325	0.3378	0.3314	0.3379	0.3225	0.3445	0.3400
0.3885	0.3215	0.3230	0.3497	0.3315	0.3368	0.3304	0.3369	0.3235	0.3455	0.3398
0.5488	0.5288	0.5124	0.5125	0.5186	0.5124	0.5124	0.5188	0.5215	0.5212	0.5206
0.5201	0.5212	0.5215	0.5188	0.5124	0.5124	0.5186	0.5125	0.5124	0.5288	0.5172
0.5255	0.5212	0.5215	0.5188	0.5124	0.5124	0.5186	0.5125	0.5124	0.5288	0.5179
0.5288	0.5212	0.5215	0.5588	0.5124	0.5524	0.5186	0.5125	0.5524	0.5288	0.5307

Table S2 Raw data for the co-efficient of variation for the intra-assay

Day 1	Day 2	Day 3	Day 4	Day 5	Day 6	Total
0.1242	0.1465	0.1012	0.1986	0.1200	0.1651	0.1471
0.314	0.314	0.321	0.331	0.314	0.3302	0.3220
0.496624	0.586	0.4048	0.79424	0.48	0.6604	0.588413
0.496624	0.586	0.4048	0.79424	0.48	0.6604	0.588413
0.496624	0.586	0.4048	0.79424	0.48	0.6604	0.588413
0.6552	0.657	0.6072	0.645	0.6541	0.6541	0.645433
0.6541	0.6541	0.645	0.6072	0.657	0.6552	0.645433
0.6521	0.652	0.6072	0.62451	0.72	0.6521	0.651318
0.6552	0.657	0.62541	0.645	0.6541	0.6541	0.648468

Table S3 Stability test for the cystatin C lateral flow strips

Cystatin-C (µg/ml)	COV %
32	1.7752
0.4	3.5032
0.015	3.5023
